# Quantification of total uncertainty in the physics-informed reconstruction of CVSim-6 physiology

**DOI:** 10.1098/rsta.2024.0221

**Published:** 2025-03-13

**Authors:** Mario De Florio, Zongren Zou, Daniele E. Schiavazzi, George Em Karniadakis

**Affiliations:** ^1^ Division of Applied Mathematics, Brown University, Providence, RI 02906, USA; ^2^ Department of Applied and Computational Mathematics and Statistics, University of Notre Dame, Notre Dame, IN 46556, USA

**Keywords:** cardiovascular physiology, physics-informed machine learning, random-projection neural networks, total uncertainty quantification, time series

## Abstract

When predicting physical phenomena through simulation, quantification of the total uncertainty due to multiple sources is as crucial as making sure the underlying numerical model is accurate. Possible sources include irreducible *aleatoric* uncertainty due to noise in the data, *epistemic* uncertainty induced by insufficient data or inadequate parameterization and *model-form* uncertainty related to the use of misspecified model equations. In addition, recently proposed approaches provide flexible ways to combine information from data with full or partial satisfaction of equations that typically encode physical principles. Physics-based regularization interacts in non-trivial ways with aleatoric, epistemic and model-form uncertainty and their combination, and a better understanding of this interaction is needed to improve the predictive performance of physics-informed digital twins that operate under real conditions. To better understand this interaction, with a specific focus on biological and physiological models, this study investigates the decomposition of total uncertainty in the estimation of states and parameters of a differential system simulated with MC X-TFC, a new physics-informed approach for uncertainty quantification based on random projections and Monte Carlo sampling. After an introductory comparison between approaches for physics-informed estimation, MC X-TFC is applied to a six-compartment stiff ODE system, the CVSim-6 model, developed in the context of human physiology. The system is first analysed by progressively removing data while estimating an increasing number of parameters, and subsequently by investigating total uncertainty under model-form misspecification of nonlinear resistance in the pulmonary compartment. In particular, we focus on the interaction between the formulation of the discrepancy term and quantification of model-form uncertainty, and show how additional physics can help in the estimation process. The method demonstrates robustness and efficiency in estimating unknown states and parameters, even with limited, sparse and noisy data. It also offers great flexibility in integrating data with physics for improved estimation, even in cases of model misspecification.

This article is part of the theme issue ‘Uncertainty quantification for healthcare and biological systems (Part 1)’.

## 1. Introduction

Characterizing uncertainty from multiple sources is crucial for developing computational models that can accurately predict real physical phenomena. However, not all sources of uncertainty are equally relevant across all applications. Consistent with the discussion in [1], common sources in scientific machine learning applications relate to the quality of *data*, assumptions in the *equations* formulated to capture physical phenomena and the *estimator* used to infer relevant states or parameters. Uncertainty from data is typically referred to as *aleatoric*, and includes the inability to precisely characterize a physical quantity due to inaccurate measurements that manifest as noise, missing or scarce data that exacerbate the ill-posed character of inverse problems and noise model misspecification. Uncertainty related to model misspecification or the effects of disregarding stochasticity is referred to as *model-form* uncertainty. In addition, for data-driven estimators, prediction variability due to the network size, hyperparameter selection and determination of optimal weights and biases in optimization or inference tasks constitute a form of *epistemic* uncertainty.

In this context, physics-informed neural networks (PINNs) [2] have emerged as widely used estimators for problems involving differential equations (DE) across diverse disciplines such as fluid mechanics [3–7], epidemiology [8–11] and beyond. Leveraging modern machine learning techniques, including automatic differentiation and efficient optimization methods, PINNs have proven effective in solving differential equations, assimilating data and tackling ill-posed inverse problems [12,13], as discussed in a number of comprehensive reviews [14,15]. Approaches derived by PINNs have also been recently applied for parameter estimation in blood flow models [16], cardiac mechanics [17] and pressure–volume dynamics in heart failure [18].

Uncertainty quantification methods for neural networks, such as variational inference [19–21], dropout [22] and deep ensemble [23], have also been integrated into PINNs for quantifying uncertainty arising from multiple sources. These include noisy and gappy data [24,25], physical model misspecification [26], noisy inputs [27] and the inherent uncertainty of neural network models [1,28,29]. However, one of the main drawbacks of PINN-based approaches is the computational cost associated with back-propagation and the need to augment the loss function to account for initial and boundary conditions, adding complexity to the process of learning DE solutions. An alternative approach, the Theory of Functional Connections (TFC) [30–34], offers a *constrained expression* to approximate differential equation solutions while analytically satisfying initial and boundary conditions. Building on this framework, the eXtreme-TFC approach (X-TFC [35]) combines TFC with random-projection neural networks (a.k.a. extreme learning machine [36,37]), providing a fast and accurate method for inference and prediction in partially observed and possibly misspecified dynamical systems. Random-projection neural networks have been widely employed in physics-informed frameworks for solving linear and nonlinear partial differential equations [37–44], inverse problems for parameter estimations and dynamical systems discovery [45–47], and neural operators [48]. While X-TFC has demonstrated robustness and efficiency in various applications [49–52], including forward problems [53–61], inverse problems for the estimation of parameters and missing terms in equations [62,63], and combined with symbolic regression for physics discovery [63,64], its uncertainty quantification capabilities have been largely overlooked in the literature. This study aims to address this gap by investigating physics-informed estimation under total uncertainty in the context of numerical models with applications in biology and physiology.

Computational models in biology have a rich history, encompassing diverse areas such as epidemiology [65], species and population dynamics [66], gene regulatory networks [67], metabolic pathways [68] and phylogenetics [69], among many others. Similarly, the field of physiology has seen a constant growth in the complexity of model formulations following early studies by Harvey [70], Poiseuille [71], Frank [72] and many others. Two- or three-element Windkessel models represent basic examples of lumped parameter haemodynamic models. These models approximate the Navier–Stokes equations in cylindrical coordinates, linearized around rest conditions [73], and are analogous to equations describing current and voltage in electrical circuits. More complex one-dimensional models [74] offer a more accurate haemodynamic representation but still rely on approximations for minor losses related, e.g. to stenosis or bifurcations. Additionally, haemodynamics can be solved using complex multi-physics three-dimensional models with fluid–structure interaction [75,76] or include cardiac electrophysiology [77,78]. In addition, a number of recent studies have focused on the quantification of uncertainty in the predictions from haemodynamic models due to variability in boundary conditions, material properties of vascular tissue [79–82] or anatomical model geometry [83]. More recent approaches have also focused on the solution of inverse problems, combined forward and inverse problems [84–87], multi-fidelity propagation and sensitivity analysis [88–92], and probabilistic neural twins [93].

The main contribution of this study is to propose a new X-TFC–based method for uncertainty quantification and to improve understanding of the interaction between total uncertainty and physics-informed regularization under a variable amount of data. We do so by focusing on time-dependent problems formulated as systems of ODEs. We begin with a controlled computational experiment to examine the behaviour of X-TFC–based estimators under separate aleatoric, epistemic and model-form uncertainties, comparing their performance with PINN and Bayesian PINN (B-PINN) estimators. Next, we conduct an ablation study for CVSim-6, a stiff differential ODE model, focusing on aleatoric and epistemic uncertainties. We assess prediction variability by systematically reducing the available data for six compartmental pressures while estimating parameters that characterize pulmonary venous resistance and aortic compliance. We then consider the common situation arising in lumped-parameter haemodynamics models, in which a compartment with linear resistance is used as a simplified model of a complex vascular tree. To study the impact of this model simplification on the total uncertainty, we introduce a discrepancy function. Furthermore, we demonstrate how different modelling choices for such discrepancy directly influence the quantification of model-form uncertainty. To the best of our knowledge, this is the first study in which the behaviour of physics-informed neural estimators is investigated under total uncertainty for stiff differential systems in the context of lumped parameter haemodynamics.

This paper is organized as follows. Section 2 introduces the CVSim-6 cardiovascular model, provides the formulation as a differential system and discusses its stiffness. Section 3 introduces the X-TFC methodology for grey-box identification, parameter estimation and how it is used for uncertainty quantification. Section 4 presents an introductory example to facilitate the reader’s understanding of total uncertainty decomposition and to better explain the individual contributions of each uncertainty source. Finally, the results for the uncertainty quantification for the CVSim-6 cardiovascular model are presented in §5, followed by a discussion and the conclusions in §6.

## 2. The CVSim-6 cardiovascular model

CVSim-6 is a lumped-parameter haemodynamic model, originally developed for teaching cardiovascular physiology [94,95]. It includes six compartments, where the subscripts l, r, a, v, pa and pv indicate quantities referred to as the left ventricle, right ventricle, systemic arteries, systemic vein, pulmonary arteries and pulmonary veins, respectively. Additionally, the subscript th is used to indicate the *transthoracic* pressure.

**Figure 1.**
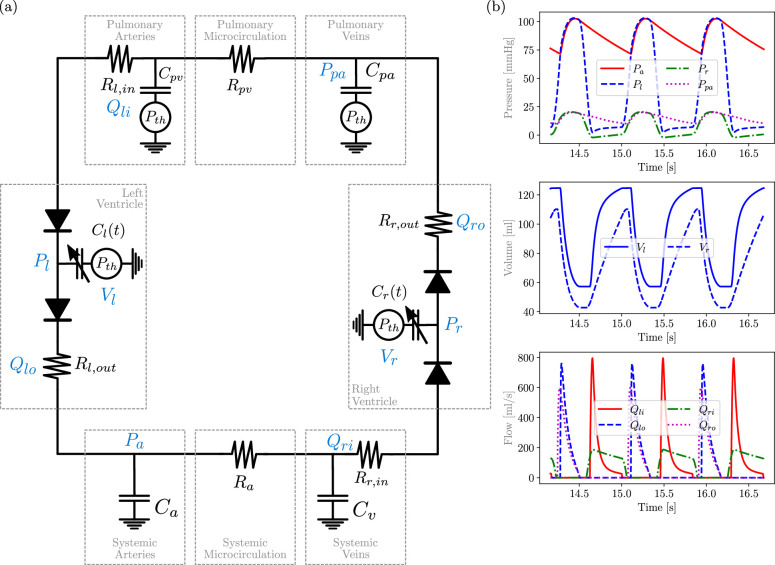
(a) CVSim-6 model circuit and (b) default output.

CVSim-6 consists of six differential equations (one per compartment) and 23 input parameters. These parameters, together with a specific combination (referred to as the *default* set) providing outputs associated with the physiology of a healthy subject, are grouped in tables 4, 5, 6 and 7 in appendix A. A schematic of the CVSim-6 model is also shown in figure 1a.

The CVSim-6 model consists of a system of six ODEs, one per compartment, expressed as


(2.1) $$\begin{array}{c} {\dot{P}}_{l}(t)=\frac{Q_{l,in}(t)-Q_{l,out}(t)-(P_{l}(t)-P_{th})\dot{C_{l}}(t)}{C_{l}(t)},\;{\dot{P}}_{a}(t)=\frac{Q_{l,out}(t)-Q_{a}(t)}{C_{a}}\\ {\dot{P}}_{v}(t)=\frac{Q_{a}(t)-Q_{r,in}(t)}{C_{v}},\;{\dot{P}}_{r}(t)=\frac{Q_{r,in}(t)-Q_{r,out}(t)-(P_{r}(t)-P_{th}){\dot{C}}_{r}(t)}{C_{r}(t)}\\ {\dot{P}}_{pa}(t)=\frac{Q_{r,out}(t)-Q_{pv}(t)}{C_{pa}},\;{\dot{P}}_{pv}(t)=\frac{Q_{pv}(t)-Q_{l,in}(t)}{C_{pv}}. \end{array}$$

Volumetric flows are defined via Ohm’s law under a unidirectional valve assumption as


(2.2) $$\begin{array}{c} Q_{l,in}(t)=\frac{P_{pv}(t)-P_{l}(t)}{R_{l,in}}I_{P_{pv}(t)>P_{l}(t)},\;Q_{l,out}(t)=\frac{P_{l}(t)-P_{a}(t)}{R_{l,out}}I_{P_{l}(t)>P_{a}(t)}\\ Q_{a}(t)=\frac{P_{a}(t)-P_{v}(t)}{R_{a}},\;Q_{r,in}(t)=\frac{P_{v}(t)-P_{r}(t)}{R_{r,in}}I_{P_{v}(t)>P_{r}(t)}\\ Q_{r,out}(t)=\frac{P_{r}(t)-P_{pa}(t)}{R_{r,out}}I_{P_{r}(t)>P_{pa}(t)},\;Q_{pv}(t)=\frac{P_{pa}(t)-P_{pv}(t)}{R_{pv}}. \end{array}$$

where the *indicator function* $I_{C}$ is equal to 1 if the condition C is true, and zero otherwise.

Finally, stressed volumes for each compartment are calculated via a linear pressure–volume relationship of the form


(2.3) $$\begin{array}{lcr} & \begin{array}{c} V_{l}(t)=V_{l}^{0}+(P_{l}(t)-P_{th})C_{l}(t),\;V_{a}(t)=V_{a}^{0}+(P_{a}(t)-\frac{1}{3}P_{th})C_{a},\\ V_{v}(t)=V_{v}^{0}+P_{v}(t)C_{v},\;V_{r}(t)=V_{r}^{0}+(P_{r}(t)-P_{th})C_{r}(t),\\ V_{pa}(t)=V_{pa}^{0}+(P_{pa}(t)-P_{th})C_{pa},\;V_{pv}(t)=V_{pv}^{0}+(P_{pv}(t)-P_{th})C_{pv}\;. \end{array} & \end{array}$$

The evolution over a few cardiac cycles for the pressures, volumes and flows associated with the default parameter set is shown in figure 1b. The values of the basic physiological quantities, capacitances, resistances and unstressed volumes used in this work are listed in appendix A.

The CVSim-6 model has two sources of nonlinearity: the unidirectional valves and the time-varying left and right ventricular capacitance, which are responsible for ventricular contraction. Both mechanisms induce stiffness—the co-existence of processes with wildly different time scales, e.g. [96]—in the mathematical solutions of the differential system, particularly during systole, when the opening of the aortic valve couples the left ventricular and systemic compartments, resulting in a particularly short relaxation time (as expressed by the equivalent RC constant). The key to achieving a correct periodic response is to carefully use implicit solvers and adaptive time-stepping.

## 3. Method

### (a) eXtreme Theory of Functional Connections

The Theory of Functional Connections (TFC) [30] provides the so-called constrained expression (CE) to approximate the solution of the differential equation in a form depending on the problem constraints [33,34,97]. Consider an initial value problem governing the evolution of the scalar quantity x∈ℝ of the form


$$\left\{ \begin{array}{ll} dx/dt & =f(x,t)\\ x(0) & =x_{0} \end{array}\right.,$$

where the unknown solution is approximated by the constrained expression [30]


$$x(t,\beta )=g(t,\beta )-g(0,\beta )+x_{0},$$

with a user-selected function g(t,β). According to the X-TFC framework [35], the function g(t,β) belongs to the family of single-layer random projection neural networks, with input weights and biases assigned randomly before training. It is expressed as


(3.1) $$g(t,\beta )=\sum_{j=1}^{L}\;\beta_{j}\sigma (w_{j}\;t+b_{j})=\;\left[\begin{array}{c} \sigma (w_{1}\;t+b_{1})\\ \sigma (w_{2}\;t+b_{2})\\ \vdots \\ \sigma (w_{L}\;t+b_{L}) \end{array}\right]\;\beta =\sigma^{T}\;\beta ,\;\;\text{and}\;\;\sigma^{T}(0)=\left[\begin{array}{c} \sigma (0)\\ \sigma (0)\\ \vdots \\ \sigma (0) \end{array}\right]=\sigma_{0}^{T},$$

where $w_{j}$, $b_{j}$ and $\beta_{j},j=1,\ldots ,L$ represent the weight, bias and output weight associated with the j-th neuron of the single hidden layer available to the network. Nonlinearity is implemented through a user-selected activation function σ(wt+b) (in this work, tanh or *softplus* activation functions are used). A parametric approximation for the solution of the original ODE [30] and its time derivative can thus be written as


(3.2) $$\begin{array}{r} x(t,\beta )={\left[\sigma -\sigma_{0}\right]}^{T}\;\beta +x_{0}, \end{array}$$

(3.3) $$\begin{array}{rl} \dot{x}(t,\beta ) & ={\dot{\sigma }}^{T}\;\beta . \end{array}$$

Black- or grey-box approximation problems are then formulated as determining the value of the coefficients β from observations of the unknown solution and/or prior knowledge of the physics (i.e. differential equation). This problem is formulated on a number of sub-domains obtained by defining n sub-intervals of equal length $h=t_{k}-t_{k-1}$, for k=1,…,n, leading to a collection of initial value problems


(3.4) $$\begin{array}{r} \left\{ \begin{array}{ll} dx^{\left(k\right)}/dt & =f(x^{\left(k\right)},t),\;\;\text{for}\;\;x^{\left(k\right)}\in [t_{k},t_{k+1}],\\ x_{0}^{\left(k\right)} & =x_{f}^{\left(k-1\right)}. \end{array}\right. \end{array}$$

where continuity of the solution on successive intervals is imposed through the boundary conditions. The interested reader is referred to the literature [35,53] for additional details.

Here, we present the X-TFC formulation used for parameter estimation in the CVSim-6 ODE system. The parameters to be estimated generate additional unknowns in each least squares solution. For improved clarity, here we present a step-by-step example based on (2.1), with unknown parameters $R_{pv}$ and $C_{a}$, and data only observed for ${\tilde{P}}_{a}$ and ${\tilde{P}}_{pa}$. We first write the constrained expressions and their time derivatives from equations (3.2) and (3.3)


(3.5) $$\begin{array}{c} P_{l}=(\sigma -\sigma_{0})\beta_{l}+P_{l_{0}},\;\;{\dot{P}}_{l}=c\dot{\sigma }\beta_{l},\;\;P_{a}=(\sigma -\sigma_{0})\beta_{a}+P_{a_{0}},\;\;{\dot{P}}_{a}=c\dot{\sigma }\beta_{a},\\ P_{v}=(\sigma -\sigma_{0})\beta_{v}+P_{v_{0}},\;\;{\dot{P}}_{v}=c\dot{\sigma }\beta_{v},\;\;P_{r}=(\sigma -\sigma_{0})\beta_{r}+P_{r_{0}},\;\;{\dot{P}}_{r}=c\dot{\sigma }\beta_{r},\\ P_{pa}=(\sigma -\sigma_{0})\beta_{pa}+P_{pa_{0}},\;\;{\dot{P}}_{pa}=c\dot{\sigma }\beta_{pa},\;\;P_{pv}=(\sigma -\sigma_{0})\beta_{pv}+P_{pv_{0}},\;\;{\dot{P}}_{pv}=c\dot{\sigma }\beta_{pv}. \end{array}$$

We can now assemble the loss function from the residuals of the six ODEs and two observed pressures $\tilde{P_{a}},\;{\tilde{P}}_{pa}$ as follows


(3.6) $$\begin{array}{c} L_{l}\equiv {\dot{P}}_{l}-\frac{Q_{l,in}-Q_{l,out}-(P_{l}-P_{th})\dot{C_{l}}(t)}{C_{l}(t)},\;L_{a}\equiv {\dot{P}}_{a}-\frac{Q_{l,out}-Q_{a}}{C_{a}},\;L_{v}\equiv {\dot{P}}_{v}-\frac{Q_{a}-Q_{r,in}(t)}{C_{v}}\\ L_{r}\equiv {\dot{P}}_{r}-\frac{Q_{r,in}-Q_{r,out}-(P_{r}-P_{th}){\dot{C}}_{r}}{C_{r}(t)},\;L_{pa}\equiv {\dot{P}}_{pa}-\frac{Q_{r,out}-Q_{pv}}{C_{pa}},\;L_{pv}\equiv {\dot{P}}_{pv}-\frac{Q_{pv}-Q_{l,in}}{C_{pv}}\\ L_{a_{data}}\equiv {\tilde{P}}_{a}-P_{a},\;L_{pa_{data}}\equiv {\tilde{P}}_{pa}-P_{pa} \end{array},$$

such that


(3.7) $$\begin{array}{lcr} & L=[L_{l}\;\;L_{a}\;\;L_{v}\;\;L_{r}\;\;L_{pa}\;\;L_{pv}{]}^{T}. & \end{array}$$

The unknown vector β is composed of the unknown output weights of the neural network and the parameters to estimate, such as


(3.8) $$\beta =[\beta_{l}\;\;\beta_{a}\;\;\beta_{v}\;\;\beta_{r}\;\;\beta_{pa}\;\;\beta_{pv}\;\;R_{pv}\;\;C_{a}{]}^{T}.$$

By computing the derivatives of the loss functions with respect to the unknowns, we get the Jacobian matrix


(3.9) $$\begin{array}{r} J=\left[\begin{array}{cccccccc} \frac{L_{l}}{\beta_{l}} & \frac{L_{l}}{\beta_{a}} & 0 & 0 & 0 & \frac{L_{l}}{\beta_{pv}} & 0 & 0\\ \frac{L_{a}}{\beta_{l}} & \frac{L_{a}}{\beta_{a}} & \frac{L_{a}}{\beta_{v}} & 0 & 0 & 0 & 0 & \frac{L_{a}}{C_{a}}\\ 0 & \frac{L_{v}}{\beta_{a}} & \frac{L_{v}}{\beta_{v}} & \frac{L_{v}}{\beta_{r}} & 0 & 0 & 0 & 0\\ 0 & 0 & \frac{L_{r}}{\beta_{v}} & \frac{L_{r}}{\beta_{r}} & \frac{L_{r}}{\beta_{pa}} & 0 & 0 & 0\\ 0 & 0 & 0 & \frac{L_{pa}}{\beta_{r}} & \frac{L_{pa}}{\beta_{pa}} & \frac{L_{pa}}{\beta_{pv}} & \frac{L_{pa}}{R_{pv}} & 0\\ \frac{L_{pv}}{\beta_{l}} & 0 & 0 & 0 & \frac{L_{pv}}{\beta_{pa}} & \frac{L_{pv}}{\beta_{pv}} & \frac{L_{pv}}{R_{pv}} & 0\\ 0 & \frac{L_{a_{data}}}{\beta_{a}} & 0 & 0 & 0 & 0 & 0 & 0\\ 0 & 0 & 0 & 0 & \frac{L_{pa_{data}}}{\beta_{pa}} & 0 & 0 & 0 \end{array}\right] \end{array}.$$

Finally, we can compute the vector of the unknowns using a least-squares algorithm to determine the k-th iterate of the coefficient vector β, which provides both the estimated parameters $C_{a}$ and $R_{pv}$, and the pressure profiles from the constrained expressions (3.5):


(3.10) $${\Delta \beta }^{k}=-{\left[J^{T}\;J\right]}^{-1}\;J^{T}\;L.$$

**Figure 2.**
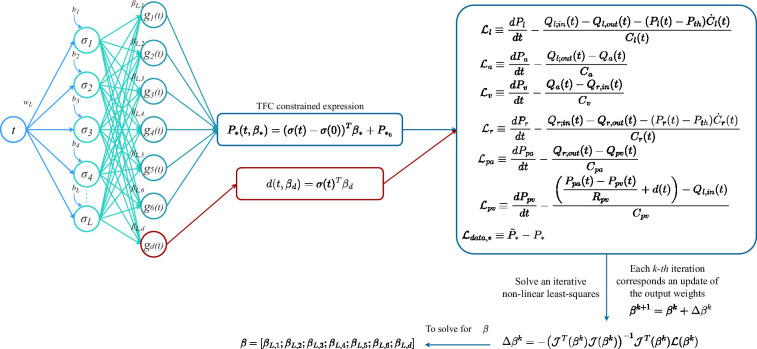
Schematic of the X-TFC algorithm for performing grey-box identification of the pulmonary flux discrepancy. Input weights and biases are randomly selected. The last step solves iteratively a least-squares problem.

Thus, substituting the coefficient vector β into the CEs and CE derivatives of (3.5), we obtain an approximation for the learned dynamics of the pressures, their derivatives in time, and as a consequence the approximation of the volumes and the flows. A representative schematic of the grey-box X-TFC algorithm, based on a loss function that only encodes model solution errors, is shown in figure 2.

As previously discussed, we use domain decomposition in time to formulate the estimation problem on small, sequentially ordered, non-overlapping subdomains. Thus, X-TFC is iteratively applied to each subdomain, selecting the initial conditions so variables are continuous across subdomain interfaces. Final parameter estimates are obtained as averages over point values obtained at each subdomain. Missing terms in the differential equations or any model discrepancies are estimated by adding a new neural network, similar to how the state variables are estimated. For more details, interested readers can refer to the grey-box X-TFC formulation in [64].

### (b) Uncertainty quantification

This section formalizes physics-informed state and parameter estimation for dynamical systems under *total uncertainty*, i.e. aleatoric, epistemic and model-form uncertainty as defined in §1. Consider a statistical model of the form


$$\tilde{x}(t)=x(t,\beta )+x_{0}+\epsilon (t),$$

where $\tilde{x}(t)$ is the underlying true process, x(t,β) is a grey-box X-TFC approximation and ϵ(t)∼N(0,C) is a heteroscedastic noise model with diagonal covariance matrix, where the square root of the diagonal elements are computed as


(3.11) $$\begin{array}{lcr} & \sigma_{i}=0.02\cdot \max_{t}(|{\tilde{x}}_{i}(t)|),\;\;\text{for}\;\;i=1,\ldots ,m, & \end{array}$$

and reported in table 1 for all CVSim-6 state variables.

**Table 1.** Noise standard deviations for pressure data.

variable	maximum value (mmHg)	$\sigma_{i}$ (mmHg)
$P_{l}$	103.6	2.07
$P_{a}$	103.0	2.06
$P_{v}$	7.3	0.14
$P_{r}$	20.4	0.41
$P_{pa}$	20.0	0.40
$P_{pv}$	12.1	0.24

Let us also assume the quantity x(t,β) to be an approximation for the solution of the system of differential equations (2.1). These equations provide only an approximation of the true circulatory response of an individual, and differ from the true response by a vector of *model-form* error components expressed as


$$\left\{ \begin{array}{l} {\dot{x}}_{1}=f_{1}(x_{1},x_{2},\ldots ,x_{m})+h_{1}(x_{1},x_{2},\ldots ,x_{m},y_{1},y_{2},\ldots ,y_{n})\\ {\dot{x}}_{2}=f_{2}(x_{1},x_{2},\ldots ,x_{m})+h_{2}(x_{1},x_{2},\ldots ,x_{m},y_{1},y_{2},\ldots ,y_{n})\\ \vdots \\ {\dot{x}}_{m}=f_{m}(x_{1},x_{2},\ldots ,x_{m})+h_{m}(x_{1},x_{2},\ldots ,x_{m},y_{1},y_{2},\ldots ,y_{n}), \end{array}\right.\;\text{or in compact form}\;\;\dot{x}=f(x)+h(x,y).\;$$

We first assume that the variables x are sufficient to describe the dynamics for the selected quantities of interest, or, in other words, h(x,y)=h(x). This leads to a modified X-TFC approximation with additional coefficients $\beta_{h}$ used to approximate the discrepancy term h, leading to the modified statistical model


(3.12) $$\tilde{x}(t)=x(t,\beta ,\beta_{h})+x_{0}+\epsilon (t),$$

which contains all three uncertainty mechanisms mentioned above.

The term ϵ(t) in (3.12) accounts for the irreducible *aleatoric* uncertainty, responsible for variability in the output of repeated model evaluations. By epistemic uncertainty, we refer to the characterization of the variability in the predicted x due to changes in the coefficients β, resulting from the random selection of the weights w and biases b in (3.1), and variability in the observed data consistent with the assumed noise model, which also informs the variability in the initial condition $x_{0}$ (perturbed using a zero-mean Gaussian noise with standard deviations in (3.11)). Additionally, *model-form* uncertainty consists of epistemic uncertainty on the discrepancy coefficients $\beta_{h}$, induced by noise in the pressure data and the random selection of the weights $w_{h}$ and biases $b_{h}$.

To quantify uncertainty in the reconstructed X-TFC response, we use a simple, scalable, yet effective Monte Carlo approach, which we refer to as MC X-TFC. Multiple instances of X-TFC are trained independently based on synthetic data with added random noise from a known distribution, each with randomly initialized weights and biases. MC X-TFC shares similarities with *deep ensembles*, as proposed in [23], which have proven to be highly effective for uncertainty quantification in neural networks, even when such uncertainty arises solely due to the random initialization of weights and biases (e.g. [1,11,26,98–103]). In the next section, we use a simple system to compare the performance of X-TFC and PINNs in the quantification of total uncertainty.

**Remark 1.** *The examples discussed in the next sections show how aleatoric, epistemic and model-form uncertainty are not independent. Aleatoric uncertainty is quantified a priori according to a known probability density and superimposed on synthetically generated data. As such, it is only affected by assumptions related to the precision of the measurement devices used to quantify blood pressures, flows and volumes. However, its volatility directly affects epistemic and model-form uncertainty in a nonlinear fashion.*

## 4. Introductory example

As an introductory example, we examine the decomposition of total prediction uncertainty produced by MC X-TFC when applied to the solution of a simple ODE system. The same decomposition is also evaluated using physics-informed neural networks, specifically the deep ensemble method for PINNs [1,11] and Bayesian PINNs [24]. In Bayesian PINNs, a posterior distribution of the network parameters is first formulated by conditioning on both data and model equations. Samples from such posterior are then generated by Hamiltonian Monte Carlo (HMC) [104], and a posterior predictive distribution is finally identified through forward network evaluations.

### (a) Decomposition of total uncertainty

Consider the following initial value problem


(4.1) $$\begin{array}{lcr} & \left\{ \begin{array}{c} \frac{dx(t)}{dt}=\frac{\cos(kt)}{k},\\ x(0)=x_{0}=10, \end{array}\right. & \end{array}$$

with exact solution $x(t)=\sin(kt)/k^{2}+x_{0}$, and k>0 is a constant. Also consider an *inverse* problem where k is unknown, and N noisy realizations of the solution x are available at irregular time intervals, resulting in a dataset $\{t_{i},x_{i}{\}}_{i=1}^{N}$. To this end, we use MC X-TFC to simultaneously perform three tasks: reconstruct x(t) from partial observations, infer the value of k from both the data and the underlying ODE (4.1) and quantify the total uncertainty using 1000 MC repetitions.

**Figure 3.**
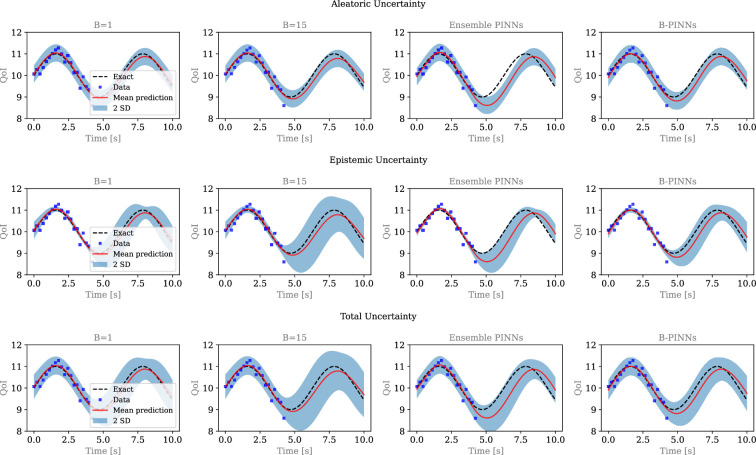
Decomposition of total uncertainty (epistemic and aleatoric) in the reconstruction of a harmonic ODE solution from noisy data using MC X-TFC. *B* denotes the bound of the uniform distribution U[−B,B] from which the input weights and biases of the hidden layer are randomly initialized. For comparison, results from ensemble PINN and B-PINN are also reported. The estimated values of *k* are presented in table 2.

The reconstructed x(t) is shown in figure 3, whereas k is estimated as 0.9840±0.0536, which agrees well with the exact value of k=1. Also, both the reconstructed MC X-TFC solution and its uncertainty are similar to those produced by the ensemble PINN and B-PINN approaches. The proposed example only considers data in [0,5), so that extrapolation for t>5 mostly relies on satisfaction of (4.1) (i.e. physics-informed regularization) using the predicted value of k. As expected, quantified epistemic uncertainty is smaller in t∈[0,5) than in t∈[5,10) due to the uneven distribution of the available data. In addition, the predicted mean of x(t) agrees well with the exact solution, and their difference is appropriately bounded by the predicted uncertainty, as shown in figure 3.

**Table 2.** Estimated values of *k* (exact value *k* = 1) and computational costs of different methods whose results are shown in figure 3. Estimates of *k* are reported as *μ ± SD*, where *μ* and *SD* are the predicted mean and standard deviation, respectively. Hyperparameters for X-TFC and PINN-based approaches can be found in Appendix B.

methods	inference of k	wall time (s)
MC X-TFC ( B=1 )	0.9840±0.0536	17.12
MC X-TFC ( B=15 )	0.9765±0.0747	5.42
ensemble ( 10 ) PINNs	0.9313±0.0317	55.28
B-PINNs	0.9595±0.0354	28.29

We also investigate how the choice of the random initialization of weights and bias in the hidden layer affects the predicted uncertainty. To do so, we fix the number of neurons in the hidden layer to m=20, and randomly draw initial choices for weights and biases from U[−B,B] using either B=1 or B=15. A larger B results in larger epistemic (and total) uncertainty, as shown in figure 3. Aleatoric uncertainty is determined by the noise model and hence irreducible, and hence is the same across different methods and/or models. Finally, the estimated value of k and a computational cost comparison between the different approaches are reported in table 2. As shown, a larger B leads to a lower wall time, since fewer least square iterations are required by the standard X-TFC algorithm, as reported in [58]. A more comprehensive ablation study of the proposed MC X-TFC in quantifying epistemic uncertainty for this example can be found in Appendix C.

**Figure 4.**
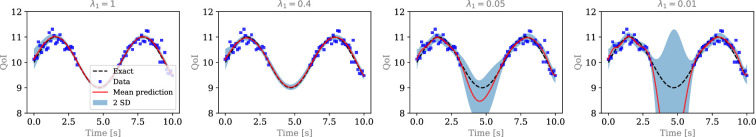
Reconstructed *x*(*t*) and epistemic uncertainty computed using MC X-TFC for a varying degree of physics-informed regularization, where $\lambda_{1}$ in (4.2) denotes the penalty coefficient in the loss function. Both the error in the predicted mean and the predicted uncertainty grow significantly as a result of increasingly relying on (missing) data as $\lambda_{1}$ is reduced.

X-TFC also allows us to control the relative amount of physics- versus data-informed regularization. To demonstrate this capability, we apply different penalty coefficients to the two main components of the loss function, i.e.


(4.2) $$\begin{array}{lcr} & L=\lambda_{1}L_{\text{eq}}+\lambda_{2}L_{\text{data}}, & \end{array}$$

where $\lambda_{1}$ and $\lambda_{2}$ are associated with the physics- and data-informed loss component, respectively. Specifically, we study the effect of a varying degree of physics-informed regularization under unevenly distributed data. When solely relying on data (small $\lambda_{1}$), the prediction error and corresponding epistemic uncertainty significantly increase, as expected, in regions where observations are missing (see figure 4).

### (b) System identification under model-form uncertainty

**Figure 5.**
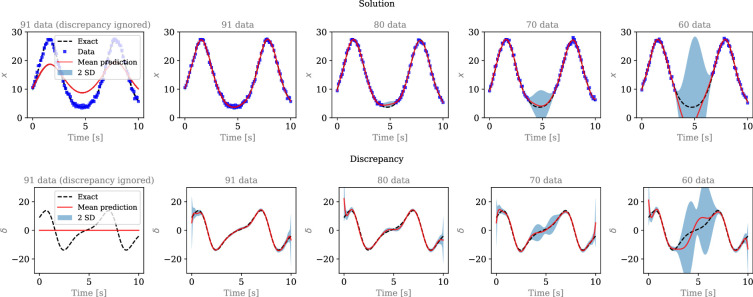
Quantification of epistemic uncertainty in the reconstructed solution of a harmonic equation from an inconsistent dataset of varying size. The first row presents an estimate for the solution x(t) while the second row represents an estimate for the discrepancy δ(t). The predicted (epistemic) uncertainty of δ(t) is used to characterize model-form uncertainty. On the leftmost plots, we present the result obtained by ignoring δ(t), and using the misspecified differential equation, hence demonstrating the use of MC X-TFC in the context of model misspecification.

In this section, we assume that the differential (4.1) is an approximation of a true underlying nonlinear model, expressed as the initial value problem


(4.3) $$\begin{array}{lcr} & \left\{ \begin{array}{c} \frac{dx(t)}{dt}=\frac{x\;\cos(kt)}{k}\\ x(0)=x_{0}=10, \end{array}\right. & \end{array}$$

which is used as the data-generating process, but whose formulation remains unknown. This scenario is a source of model-form uncertainty. We deal with this situation by modifying (4.1) with the addition of an unknown discrepancy, which is learnt, as discussed in §3a and figure 2, to compensate for the inconsistency between the linear and nonlinear models, such as


(4.4) $$\begin{array}{lcr} & \left\{ \begin{array}{c} \frac{dx(t)}{dt}=\frac{\cos(kt)}{k}+\delta (t)\\ x(0)=x_{0}=10. \end{array}\right. & \end{array}$$

We note that the exact solution is $x(t)=x_{0}\exp[(1/k^{2})\;\sin(kt)]$ and hence the discrepancy can be computed exactly as δ(t)=(x(t)−1)[cos⁡(kt)/k].

Results are presented in figure 5, where the reconstructed x(t) and discrepancy δ(t) are shown in the first and second row, respectively, together with the quantified uncertainty. On the leftmost plot in figure 5, we present the consequence of utilizing the misspecified model directly (with k being unknown and learnable) while ignoring the discrepancy, i.e. δ(t)=0,∀t∈[0,10]. As shown, MC X-TFC fails to fit the data, as the underlying equation does not agree with the available observations. Modelling the discrepancy δ(t) with an additional network allows the equation loss $L_{\text{eq}}$ and the data loss $L_{\text{data}}$ to be simultaneously minimized, so that the data of x are fitted and the (corrected) equation is satisfied [26]. From figure 5, we can see that the discrepancy is also accurately captured when irregularly sampled and noisy data are available. As the number of data decreases, the accuracy of both the reconstructed x(t) and inferred δ(t) is reduced, and the errors between their predicted mean and true solution are bounded by the predicted uncertainties. We note that in this case, we fix k=1 to avoid solution multiplicity brought by the unknown parameter k and unknown discrepancy δ(t). This is a modelling choice that interacts with the quantification of model-form uncertainty. This aspect will be further discussed in §5b.

## 5. Results for the CVSim-6 cardiovascular model

We consider two applications of MC X-TFC to the CVSim-6 cardiovascular system. The first consists of an *ablation* study, where we are interested in determining how much data are needed for MC X-TFC to accurately estimate states related to synthetically generated time histories of blood pressure, flow and volume while, at the same time, estimating a pulmonary resistance and a systemic compliance parameter. In addition, we would like to quantify the total uncertainty (aleatoric plus epistemic) associated with these predictions.

The second application focuses on model-form uncertainty, particularly fitting data with an inadequate model. This situation arises very often with lumped parameter models in haemodynamics, for example, when using perfect unidirectional valves without accounting for possible regurgitation [105], when neglecting flow contributions from collateral flow, or when excluding atria or organ-level compartments from the model.

### (a) Ablation study under combined aleatoric and epistemic uncertainty

The MC X-TFC framework allows to naturally combine information from the available data and the CVSim-6 model equations. Therefore it offers an ideal testbed for determining the minimum amount of data needed to estimate states or parameters of a given system, and also to quantify the uncertainty associated with these estimates. To demonstrate this process in the context of computational physiology, we perform an ablation study where pressure data are progressively removed under physics-informed regularization, and first one and then two parameters are simultaneously estimated. We then report the estimated pressure, flow and volume traces and their variability under combined aleatoric and epistemic uncertainty. This analysis is conducted across six scenarios, as outlined in table 3, and considers data acquired on six pressures and two parameters—the pulmonary venous resistance and the systemic arterial compliance—since they are clinically relevant in the assessment of cardiovascular function.

**Table 3.** Matrix of measurements and parameters used in the ablation study.

type	Qty	Sc1	Sc2	Sc3	Sc4	Sc5	Sc6
measurements	$P_{l}$	✓	✓	✗	✗	✗	✗
	$P_{a}$	✓	✓	✓	✓	✓	✓
	$P_{v}$	✓	✓	✓	✓	✗	✗
	$P_{r}$	✓	✓	✓	✗	✗	✗
	$P_{pa}$	✓	✓	✓	✓	✓	✓
	$P_{pv}$	✓	✗	✗	✗	✗	✗
parameters	$R_{pv}$	✗	✗	✗	✗	✗	✗
	$C_{a}$	✓	✓	✓	✓	✓	✗

#### (i) State and parameter estimation under total uncertainty

**Figure 6.**
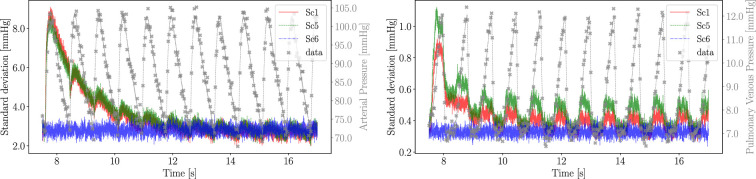
Standard deviations of estimated systemic arterial (left plot) and pulmonary venous (right plot) pressures for three scenarios (Sc1, Sc5, Sc6) under aleatoric and epistemic uncertainty are shown in red, green and blue lines, with values reported on the left y-axis scale. Noisy data and mean pressure data values are instead drawn in grey markers and lines, respectively, with values indicated on the right y-axis.

Figure 6 shows the mean and standard deviation for the estimated systemic arterial and pulmonary venous pressures under total uncertainty. After a few cardiac cycles, the Monte Carlo standard deviation of the two pressures reduces to approximately 3.0 and 0.5 mmHg for $P_{a}$ and $P_{pv}$, respectively, with values that are only marginally affected by the difference of data availability across scenarios. Knowledge of the correct underlying equations allows MC X-TFC to identify the system’s response under limited uncertainty, even when simultaneously estimating unknown parameters. However, the loss of information created by the missing parameters needs to be compensated by providing pressure data on the same compartments (systemic and pulmonary, respectively).

It is also evident from figure 6 that the amount of variability reduces with time before reaching a periodic behaviour for all scenarios except Scenario 6. This is the result of a *filtering* process due to physics-informed regularization of random initial conditions, as suggested by equation (3.4). In other words, for Sc1–Sc5, the CVSim-6 equations are alone sufficient to reconstruct the physiological response, and availability of noisy data provides redundant information that is *distilled* over time. This is confirmed by the smooth time history of the standard deviation for the pressure state variables under epistemic uncertainty. When instead two parameters are estimated in Sc6, pressure data become essential to the estimation process, as confirmed by the more *noise-like* time history for the standard deviation under epistemic uncertainty. In summary, the transition between Sc5 and Sc6 represents a switch from an estimation process where the physics and data *compete*, to a process where the physics and data *cooperate*.

**Figure 7.**
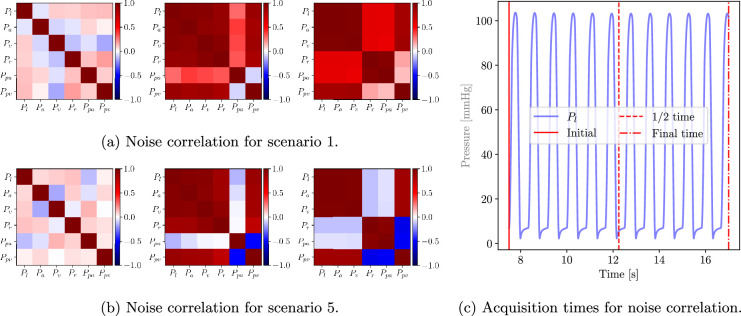
Time snapshots of correlation matrices for pressure unknowns. Snapshots are shown for (a) scenario 1 and (b) scenario 5 at initial, 1/2 and final times (left to right). (c) Selection of initial, 1/2 and final times for pressure correlation snapshots.

To further understand how the statistical structure of the noise is affected by this *filtering* process, the noise correlation matrix for all compartmental pressures is also shown in figure 7. Correlations are representative of three solution snapshots, at the beginning, half-time and final simulation time, which correspond to early systole, diastole and systole, respectively. Correlation matrices are shown for Scenarios 1 and 5 in figure 7a,b, respectively. The first snapshot confirms that noise in the initial condition is independently applied on each pressure. The second and third snapshots (see figure 7c) show a high correlation between all pressure components except $P_{pa}$ in diastole and ($P_{pa}$, $P_{r}$) in systole. The smooth pressure reconstruction achieved by X-TFC results in highly correlated noise among different pressure components, facilitated by the communication between compartments following valve openings. By contrast, the lack of correlation between $P_{pa}$ and $P_{r}$ is attributed to variability in the $R_{pv}$ parameter, which fluctuates as estimates are updated over time. Unlike Sc1 and Sc5, in Sc6 the noise in different pressure components remains uncorrelated over time (not shown).

**Figure 8.**
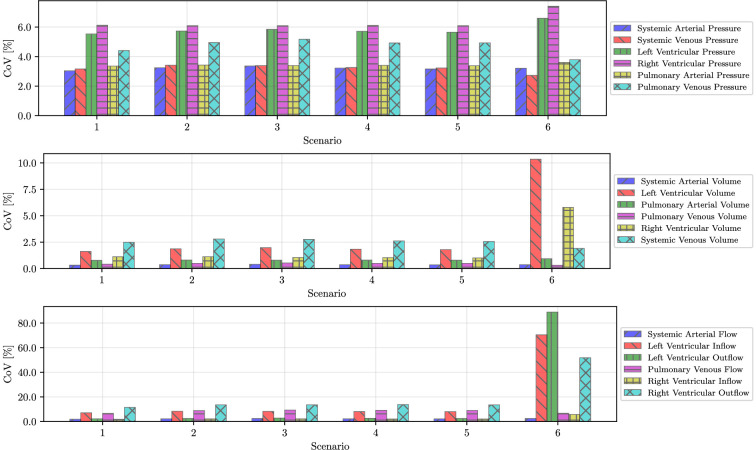
Ablation study summary. Time-average coefficients of variations (last two cardiac cycles) for pressure (top), volume (centre) and flow (bottom) quantities of interest and all scenarios.

As reported in figure 8, in Scenarios 1 to 5, the CoV is approximately 3% for $P_{a}$, $P_{v}$ and $P_{pa}$, 6% for $P_{l}$ and $P_{r}$ (due to higher measurement noise) and 5% for $P_{pv}$, as a result of the estimation of $r_{pv}$ in Sc1 to Sc5. CoV is instead below 2% for all volumes except for pulmonary veins, where it is slightly above 2.5%. Flow uncertainty is sensibly higher, with CoV for the systemic flow (i.e. systemic arterial flow, left ventricular outflow and right ventricular inflow) equal to approximately 2.5%, 8.5% for the pulmonary flow CoV, except for the right ventricular outflow, where it is approximately 13.5%. Since flows are estimated by minimizing a residual that contains derivatives of a pressure approximated from noisy observations, this higher variability is expected.

A regime shift is observed for Sc6, where the arterial compliance $c_{a}$ is also estimated as part of the solution process. In this scenario, the uncertainty doubles for the left and right ventricular pressures and increases substantially for the left ventricular volume, inflow and outflow. Satisfaction of noisy pressure measurements on the systemic pressure (remember that only $P_{a}$ and $P_{pa}$ are observed in this scenario) can be realized by either changing the left ventricular volume or the aortic compliance (whose estimated value changes with time). If this was a Bayesian estimation problem, we would say that the posterior marginal of left ventricular volume and aortic compliance shows a negative correlation due to a lack of identifiability from pressure data.

**Figure 9.**
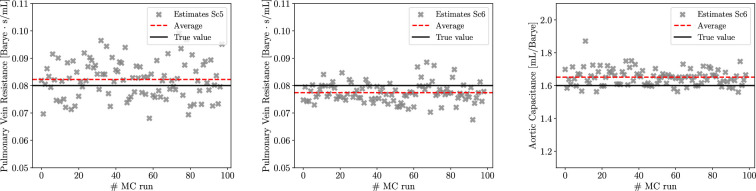
Estimates of pulmonary resistance and aortic compliance for Scenarios 5 and 6.

We also investigate the ability of MC X-TFC to estimate physiologically relevant parameters. Parameter estimates are computed on a per-simulation basis and averaged over a number of Monte Carlo runs, as shown in figure 9. Final averages agree well with true parameter values, with relative per cent errors for $R_{pv}$ equal to 1.16%, 4.74%, 3.81%, 4.11%, 2.79% and 3.24% for Sc1 to Sc6, respectively. Additionally, in Sc6, the relative per cent error for $C_{a}$ was found equal to 3.19%.

**Figure 10.**
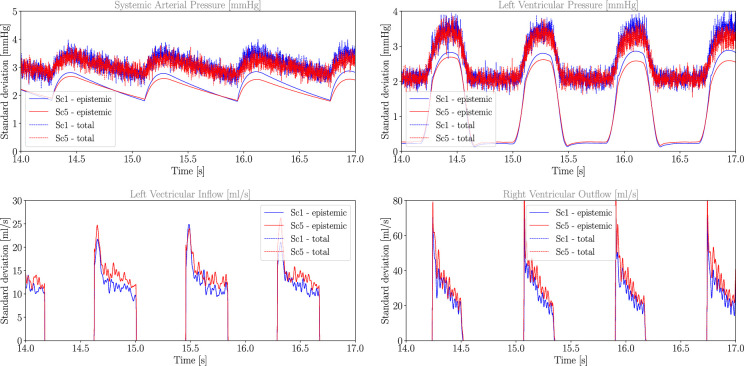
Decomposition of total uncertainty in Sc1 and Sc5 for selected pressure and flow quantities of interest (QoIs). For demonstration purposes, we have assumed the possibility of measuring flow with infinite precision, resulting in negligible aleatoric uncertainty.

Finally, we analyse how total uncertainty can be decomposed into its aleatoric and epistemic components, considering only Scenarios 1 and 5. Figure 10 illustrates this decomposition for $P_{a}$, $P_{l}$, $Q_{li}$ and $Q_{lo}$. The oscillating nature of the total uncertainty is consistent with its calculation as a Monte Carlo estimate based on 100 samples. For systemic arterial and left ventricular pressures, the uncertainty is mostly epistemic, except at diastole, where the signal-to-noise ratio is smaller. Flow and volume quantities of interest are estimated from MC X-TFC as a result of the CVSim-6 equations and observed data. As a result, any characterization of aleatoric uncertainty comes directly from assumptions about the precision of the tool or device used to measure such quantities. In figure 10, we highlight this by assuming infinite precision, causing epistemic and total uncertainty to coincide. In other words, aleatoric uncertainty in *derived* variables (e.g. blood flows and volumes) does not affect the estimation process, and can be added in post-processing.

The results for all scenarios are obtained with subdomains of length 0.001 s, five collocation points per subdomain and five neurons. With this set-up, the computational time is about 6 s per MC realization and approximately 10 min for quantifying total uncertainty through 100 MC repetitions. Therefore, the fast execution times for MC X-TFC and the fact that no offline training is required make it ideal for online state and parameter estimation.

### (b) Misspecified compartment: linear approximation of nonlinear pulmonary resistance

In this section, we investigate an important aspect of simulating circulatory systems with lumped parameter models, which typically provide an oversimplified representation of the physiological response based on linear resistor, inductor, capacitor circuits, ideal unidirectional valves, and that often selectively consider the presence of organ-level compartments, depending on the application. With reference to the CVSim-6 system, we consider a specific equation from (2.2) governing flow in the pulmonary compartment


(5.1) $$Q_{\text{lin},pv}(t)=\frac{P_{pa}(t)-P_{pv}(t)}{R_{pv}},$$

and use all the six pressures as available data. Due to the large number of branches that typically characterize the pulmonary arterial tree, we consider this linear relation to be an approximation of the more accurate nonlinear one


(5.2) $$\begin{array}{lcr} & Q_{\text{nonlin},pv}(t)=\frac{P_{pa}(t)-P_{pv}(t)}{R_{pv}(Q_{pv})}, & \end{array}$$

where a nonlinear resistance is added, which accounts for the larger contribution of minor pressure losses at bifurcations for an increasing pulmonary flow (see figure 1). To account for the difference between a linear and nonlinear pressure-flow behaviour, we use the flexibility of MC X-TFC to add a *discrepancy* term δ(t,β) to (5.1), such that


(5.3) $$Q_{\text{disc},pv}(t)=\frac{P_{pa}(t)-P_{pv}(t)}{R_{pv}}+\delta (t,\beta ),$$

and estimate this term from the available pressure data plus the satisfaction of the CVSim-6 differential equations, by approximating it with another neural network, such as


(5.4) $$\delta (t,\beta )=\sigma \beta_{\delta }.$$

Then, we verify the capability of MC X-TFC to correctly estimate the pulmonary flow discrepancy under deterministic conditions and both aleatoric and epistemic uncertainty. In the following, we will refer to equations (5.1)–(5.3) as the *linear*, *nonlinear* and *linear+discrepancy* model, respectively.

**Figure 11.**
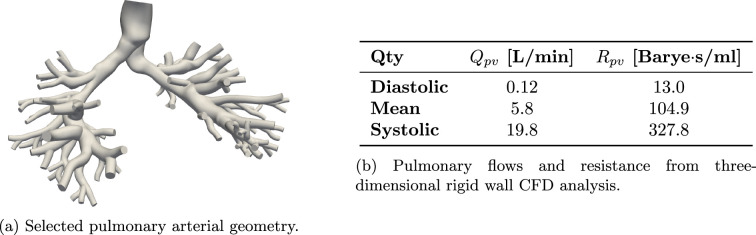
Determination of nonlinear pulmonary resistance (b) from a three-dimensional arterial tree model (a).

The values of linear and nonlinear resistance for this application are selected with reference to the pulmonary arterial anatomy shown in figure 11a. In figure 11b, we report the equivalent model resistance under mean, systolic and diastolic flow, where the strong dependence of resistance from flow is evident. Note how the resistance under mean flow is very similar to the value of $R_{pv}$ in table 6. Therefore, we consider a linear model with the default resistance $P_{pv}$ in table 6, and use linear interpolation to determine a flow-dependent resistance to be used in the nonlinear model.

**Figure 12.**
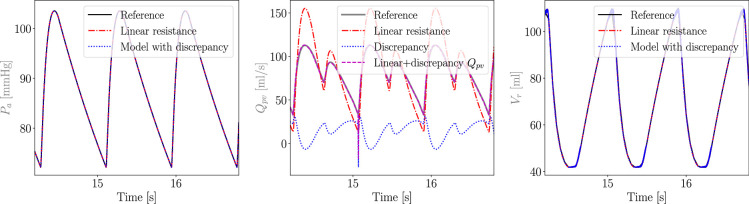
Difference in arterial pressure (left), pulmonary venous flow (centre) and right ventricular volume (right) between the true nonlinear model, and linear model with and without discrepancy, under ideal noiseless data. The plots on the left and right show how the use of a misspecified linear resistor only affects flow and pressure in the pulmonary compartment (we only show $P_{a}$ and$V_{r}$ but all other non-pulmonary flow, pressures and volumes remain the same), under noiseless data synthetically generated by the true underlying nonlinear model. As shown in the central figure, the flow estimated by a misspecified linear model overestimates the true systolic flow as a result of considering a constant rather than flow-dependent pulmonary resistance. The central figure also shows how the discrepancy learned by X-TFC correctly restores the correct pulmonary flow.

We then investigate the ability of MC X-TFC to recover the correct discrepancy under ideal noiseless measurements and complete knowledge of the CVSim-6 equations. We generate ideal data from the true nonlinear model and reconstruct the physiological response using the linear and discrepancy models. The resulting difference in arterial pressure, pulmonary venous flow and right ventricular volume between these three models is illustrated in figure 12. Since the linear resistance was selected equal to the resistance at mean flow, and the linear and nonlinear models have identical capacitance, the difference in response between the two models remains confined to the pulmonary venous compartment. Therefore, under ideal noiseless conditions, X-TFC correctly recovers the flow discrepancy, as shown in figure 12.

**Figure 13.**
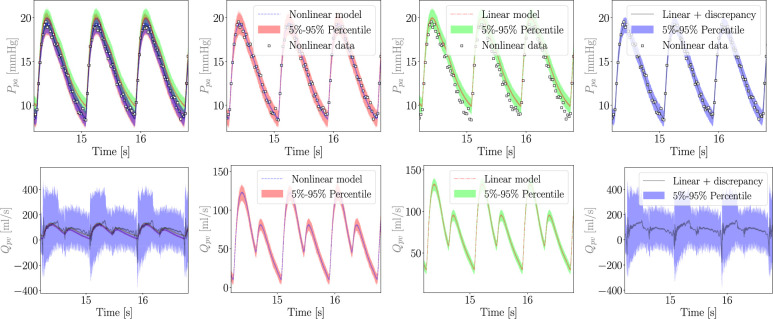
Reconstructed pulmonary arterial pressure (top) and pulmonary venous flow (bottom) under total uncertainty, for true nonlinear (second column), misspecified linear (third column) and linear plus discrepancy (right) model formulations. The first column shows a superposition of all average reconstructions and associated 5–95% confidence regions, whereas the same reconstructions and confidence regions are shown separately in the other columns for improved clarity. For the pressure predictions shown in the top row, the available data are also plotted using white squared markers. Pressure bias due to a misspecified linear pulmonary resistance (top row) is significantly reduced with the addition of a discrepancy term as shown in (5.3). However, stiffness in the CVSim-6 ODEs and lack of inertia result in large confidence intervals and even non-physiological negative pulmonary flow (bottom row).

We now consider MC X-TFC reconstruction under total uncertainty, including model-form uncertainty. Results for pulmonary arterial pressure and pulmonary venous flow are reported in figure 13. While no bias was observed for the linear model under ideal conditions, adding noise to the pressure observations induces bias consisting of a moderate increase in the pulmonary arterial pressure for the linear model. However, once a discrepancy is introduced and estimated, the bias is practically eliminated, and the total uncertainty in the pressure almost coincides with the uncertainty of the true nonlinear model. Conversely, the mean estimated pulmonary flow shows significant oscillations and is associated with substantial total uncertainty, even non-physical negative values. This is due to two factors: the first relates to modelling assumptions in the CVSim-6 model, which lacks any form of inertance, allowing sudden variations in flow to go unopposed by the system. The second relates to the regularity of the flow discrepancy, which is governed by the equation of a capacitor, which includes the derivative of pressure reconstructions for $P_{pa}$ and $P_{pv}$ that are affected by significant oscillations due to the stiffness of CVSim-6 at systole. In other words, modelling assumptions combined with the specific choice of the variable chosen to represent the discrepancy directly affect the quantification of model-form and total uncertainty.

**Figure 14.**
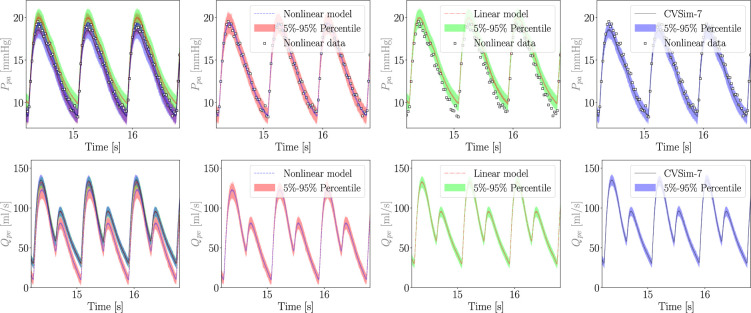
Reconstructed pulmonary arterial pressure (top) and pulmonary venous flow (bottom) under total uncertainty, for true nonlinear (second column), misspecified linear (third column) and linear plus discrepancy (right) model formulations for the system with additional inertia. The first column shows a superposition of all average reconstructions and associated 5–95% confidence regions, whereas the same reconstructions and confidence regions are shown separately in the other columns for improved clarity. For the pressure predictions shown in the top row, the available data are also plotted using white squared markers. Pressure bias due to a misspecified linear pulmonary resistance (top row) is still reduced with the addition of a discrepancy term, this time formulated as an inductance equation in (5.5). Additionally, a more physiologically consistent discrepancy formulation greatly reduces total uncertainty in pulmonary flow (bottom row).

A simple remedy is to add an inductor equation for the flow discrepancy of the form


(5.5) $$\begin{array}{lcr} & \dot{\delta }(t)=\frac{P_{pa}(t)-P_{pv}(t)}{L_{pv}}, & \end{array}$$

where $L_{pv}$ represents the inductance of the pulmonary flow discrepancy, for this case chosen as $L_{pv}=10\;r_{pv}$, and the δ(t) is now approximated via a new TFC constrained expression as


(5.6) $$\delta (t,\beta )=(\sigma -\sigma_{0})\beta_{\delta }+\delta_{0},$$

where the initial condition $\delta_{0}$ is arbitrarily set to 0. This addresses two problems at the same time by both adding inertia and, therefore, improving the realism of the CVSim-6 model, and posing additional conditions on the regularity of the flow discrepancy. We refer to this new model configuration as *CVSim-7* due to the additional differential equation introduced for the discrepancy. The results are shown in figure 14, where the discrepancy is able to reduce the bias in $P_{pa}$ while producing a physiologically consistent pulmonary venous flow.

The results for the discrepancy models are obtained with subdomains of length 0.01 s, 10 collocation points per subdomain and 10 neurons. Computing the average discrepancy requires a computational time of about 6 s per MC realization, totalling about 10 min for the whole simulation and total uncertainty quantification for 100 MC realizations. Again, MC X-TFC confirms its robustness and efficiency for online estimation under model-form uncertainty and does not require offline training.

## 6. Discussion and conclusions

This study focuses on characterizing aleatoric, epistemic and model-form uncertainty in the physics-informed reconstruction of synthetic responses generated by ODE systems. We first illustrate the performance of PINNs, B-PINNs and MC X-TFC on a single-equation differential model where the solution is reconstructed under total uncertainty, i.e. combining aleatoric, epistemic and model-form uncertainty. We then use MC X-TFC to estimate the physiologic response of the CVSim-6 compartmental model, first considering aleatoric and epistemic uncertainty in an ablation study, and then adding model-form uncertainty to consider misspecification in the pulmonary compartment. MC X-TFC produces accurate reconstructions with limited uncertainty (up to approx. 3 mmHg on systemic arterial pressure, and generally smaller than variability in the clinical assessment of pressures, flows and volumes), with fast execution and without requiring offline training.

By progressively removing data and estimating an increasing number of parameters under a correctly formulated model, we observe the transition from a competitive to a cooperative interaction between data-informed and physics-informed approximation of the true underlying ODE solution. In addition, we have shown that the specific formulation of an unobserved discrepancy term, introduced to compensate for model misspecification, strongly affects model-form uncertainty. While this issue can be mitigated by adding data, we chose to provide additional regularization through physics by adding an inductance, which injects inertia to the CVSim-6 system, while also posing additional conditions on the rate of change of the pulmonary flow. This reduces estimation bias in the pulmonary arterial pressure, and also results in a physiologically sound estimate of the pulmonary flow.

It is important to note that this study focuses on the online reconstruction of an ODE solution based on equations and data, differing from other parameter estimation approaches that rely on offline processes. In those approaches, offline work is used to learn the forward or inverse map between model parameters and outputs, or to establish prior or posterior information that informs the online process. This distinction is evident from the results of Sc6 in our ablation study. When two parameters need to be estimated jointly, the information removed from the system must be compensated by data from co-located compartments to ensure the underlying physical response is well-defined. The independence between compartments and the lack of inertia in the CVSim-6 equations seem to limit the number of parameters that can be estimated from a given dataset, relying on correlations between parameters or redundancies in model components. This observation suggests potential future work in extending X-TFC with a hybrid offline/online estimation approach.

We would also like to emphasize how the methods discussed in this paper are broadly applicable to online estimation under uncertainty for a wide variety of dynamical systems such as nuclear reactor dynamics, chemical kinetics, systems biology and chaotic systems, as widely shown in the literature [55,60,63,64]. For new dynamical systems, the physics losses and the Jacobian matrix (3.9) need to be set up accordingly. Aleatoric and epistemic uncertainty are treated exactly in the same fashion, with results that are affected by the amount of measurement uncertainty and noise in time series data for the specific application. The proposed approach is extremely flexible for model-form uncertainty, allowing one to implement discrepancies and enforce the satisfaction of additional physics.

While this study focuses on understanding the interaction between physics-informed regularization and total uncertainty for compartmental models in physiology, several limitations may restrict the ability to generalize these findings to more realistic conditions. First, we consider a Gaussian noise model on top of synthetically generated data instead of real patient-specific data. Second, some of the pressure measurements considered available in our study would be difficult to continuously measure in patients, or would only be available as time statistics, e.g. as mean, systolic or diastolic values. Third, the CVSim-6 is a relatively simple model for the human physiology and does not account for a number of important physiological mechanisms, including a four-chamber heart model, cardiorespiratory coupling or pressure autoregulation mechanisms. Finally, the range of model parameters considered in this study relate to healthy conditions in human adults, but the model and X-TFC estimation process could be adapted to specific pathological conditions and measurement scenarios.

Future work will focus on using MC X-TFC to estimate a larger number of physiologically relevant parameters, more complex models, or on applications to ICU patients, where clinical data are continuously acquired from multiple sensors. In such a scenario, fast online estimation informed by continuously acquired clinical signals is crucial for enhancing model-based patient monitoring and assessing critical conditions.

## Figures and Tables

**Figure 15.**
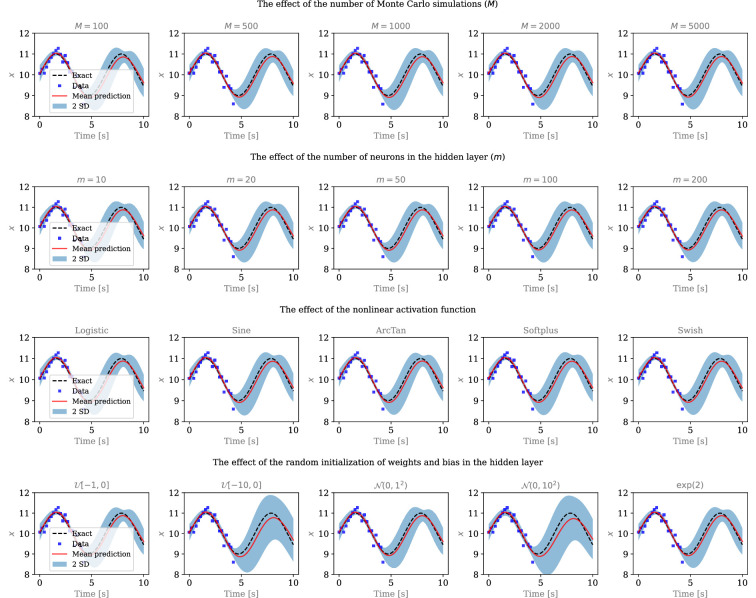
Ablation study of MC X-TFC in quantifying epistemic uncertainty for the harmonic ODE solution in §4a. Here *M* denotes the number of Monte Carlo simulations, *m* denotes the number of neurons in the hidden layer of X-TFC, U denotes the 1D uniform distribution, N denotes the one-dimensional Gaussian distribution and exp⁡(μ) denotes the exponential distribution with mean *μ*.

## Data Availability

Codes and data are available at the following Zenodo repository link: [106].

## Appendix A. Default parameters from the CVSim-6 cardiovascular model

This section provides a list of default CVSim-6 model parameters, including basic parameters (table 4), and values of capacitance (table 5), resistance (table 6) and unstressed volume (table 7), corresponding to a healthy physiological response.

**Table 4.** Basic physiological quantities.

num.	description	ref.	unit
1.	heart rate ( Hr )	72.00	(bpm)
2.	transthoracic pressure ( $P_{th}$ )	−4.00	(mmHg)
3.	systolic ratio per cardiac cycle ( $r_{sys}$ )	0.33	−

**Table 5.** Capacitances of each compartment.

num.	description	ref.	unit
4.	left ventricular diastolic capacitance ( $C_{l,dia}$ )	$7.50\cdot 10^{-3}$	(ml/Barye)
5.	left ventricular systolic capacitance ( $C_{l,sys}$ )	$3.00\cdot 10^{-4}$	(ml/Barye)
6.	arterial capacitance ( $C_{a}$ )	$1.20\cdot 10^{-3}$	(ml/Barye)
7.	venous capacitance ( $C_{v}$ )	$7.50\cdot 10^{-2}$	(ml/Barye)
8.	right ventricular diastolic capacitance ( $C_{r,dia}$ )	$1.50\cdot 10^{-2}$	(ml/Barye)
9.	right ventricular systolic capacitance ( $C_{r,sys}$ )	$9.00\cdot 10^{-4}$	(ml/Barye)
10.	pulmonary arterial capacitance ( $C_{pa}$ )	$3.23\cdot 10^{-3}$	(ml/Barye)
11.	pulmonary venous capacitance ( $C_{pv}$ )	$6.30\cdot 10^{-3}$	(ml/Barye)

**Table 6.** Resistance of each compartment (outflow resistance equal to the inflow resistance of the following compartment).

num.	description	ref.	unit
12.	left ventricular input resistance ( $R_{l,in}$ )	13.33	(Barye ⋅ s/ml)
13.	left ventricular output resistance ( $R_{l,out}$ )	8.00	(Barye ⋅ s/ml)
14.	arterial resistance ( $R_{a}$ )	1333.22	(Barye ⋅ s/ml)
15.	right ventricular input resistance ( $R_{r,in}$ )	66.66	(Barye ⋅ s/ml)
16.	right ventricular output resistance ( $R_{r,out}$ )	4.00	(Barye ⋅ s/ml)
17.	pulmonary venous resistance ( $R_{pv}$ )	106.66	(Barye ⋅ s/ml)

**Table 7.** Unstressed volume of each compartment.

num.	description	ref.	unit
18.	unstressed left ventricular volume ( $V_{l}^{0}$ )	15.00	(ml)
19.	unstressed arterial volume ( $V_{a}^{0}$ )	715.00	(ml)
20.	unstressed venous volume ( $V_{v}^{0}$ )	2500.00	(ml)
21.	unstressed right ventricular volume ( $V_{r}^{0}$ )	15.00	(ml)
22.	unstressed pulmonary arterial volume ( $V_{pa}^{0}$ )	90.00	(ml)
23.	unstressed pulmonary venous volume ( $V_{pv}^{0}$ )	490.00	(ml)

## Appendix B. Details of methods compared in the introductory example

This section provides details of methods (the MC X-TFC method, the ensemble PINNs method and the B-PINNs method) compared in §4. One thousand Monte Carlo simulations were performed for the MC X-TFC method, 10 PINNs were independently trained in the ensemble PINNs method, and 1000 posterior samples were taken in HMC along with 2000 burn-in samples for a satisfactory acceptance rate [11,24] in the B-PINNs method. The open-source NeuralUQ library [11] was used for fast and convenient implementations of HMC in B-PINNs. We note that although in this work, the training of X-TFC and PINNs was conducted sequentially, both the MC X-TFC and the ensemble PINNs methods are able to be accelerated by leveraging advanced vectorization and/or parallelization techniques for fast uncertainty quantification [1]. In the MC X-TFC method, the width of the hidden layer was set to 20, while in the ensemble PINNs and the B-PINNs methods, the neural network was chosen to have one hidden layer with 50 neurons for acceptable results. The activation function was set to the hyperbolic tangent in all methods. In the training of PINNs, the Adam optimizer [107] with $1\times 10^{-3}$ was employed for 30 000 iterations. The comparison was performed on a standard laptop CPU (13th Gen Intel(R) Core(TM) i9-13900HX with 2.20 GHz processor).

## Appendix C. Ablation study of MC X-TFC in quantifying epistemic uncertainty for the harmonic ODE solution

In this section, we conduct an ablation study of the proposed method, i.e. MC X-TFC, in quantifying epistemic uncertainty for the example presented in §4a. Specifically, we investigate the effect of the number of Monte Carlo simulations (denoted as M), the number of neurons in the hidden layer (denoted as m), the nonlinear activation function and the initialization method of the weights and biases in the hidden layer. Here we investigate five activation functions (apart from the hyperbolic tangent): logistic function, sine function, inverse tangent function, Softplus activation function and Swish activation function [108]. The baseline parameters used in all the examples in the paper are M=1000, m=20, hyperbolic tangent and the uniform distribution on [−1,1] (denoted as U[−1,1]), unless stated otherwise.

As shown in the first three rows of figure 15, results of MC X-TFC are consistent across different numbers of Monte Carlo simulations, numbers of neurons in the hidden layer and activation functions. The fourth row of figure 15 presents results of MC X-TFC with the following five random initialization distributions: the uniform distribution U[−1,0], the uniform distribution U[−10,0], the normal distribution $N(0,1^{2})$, the normal distribution $N(0,10^{2})$ and exp⁡(2), where exp⁡(μ) denotes the exponential distribution with mean μ. We note that the random initialization distribution U[−1,0] only yields non-positive weights and biases in the hidden layer, while exp⁡(2) generates only strictly positive values. Nonetheless, they are able to produce results similar to the one with U[−1,1] (presented in figure 3), demonstrating that MC X-TFC is robust to the choice of random initialization. However, we observe that the predicted epistemic uncertainty is sensitive to the scale of randomly initialized weights and biases, which is consistent with results presented in §4a, i.e. a larger scale leads to a larger epistemic uncertainty.
